# Investigation of the In Vitro Antioxidant, Anticholinesterase, Antiurease, Antityrosinase, and Cytotoxic Properties of a Novel Compound: 4‐Methoxy‐2‐(4‐Methoxyphenyl)Benzo[d][1,3,2]Dioxaborole

**DOI:** 10.1002/prp2.70044

**Published:** 2025-01-09

**Authors:** Hamdi Temel, Emine Baydan

**Affiliations:** ^1^ Department of Pharmacology, Faculty of Medicine Yozgat Bozok University Yozgat Turkey; ^2^ Department of Pharmacology and Toxicology, Institute of Health Sciences Ankara University Ankara Turkey; ^3^ Department of Pharmacology and Toxicology, Faculty of Veterinary Ankara University Ankara Turkey

**Keywords:** 3‐methoxy catechol, 4‐methoxy phenyl boronic acid, antioxidant activities, antiurease and antityrosinase activities, cytotoxic effects

## Abstract

In this study, the structure of a new boron compound obtained using 3‐methoxy catechol and 4‐methoxy phenyl boronic acid was characterized by ^1^H, ^13^C NMR, LC‐MS‐IT‐TOF, UV‐Vis and FTIR spectroscopy. The antioxidant activities of the newly synthesized compound were evaluated by DPPH free radical scavenging, ABTS quation radical scavenging and CUPRAC copper reducing capacity methods. Anticholinesterase activities were determined by acetylcholinesterase and butyrylcholinesterase enzyme inhibitor assays. Antiurease and antithyrosinase enzyme inhibition activities were also examined. Cytotoxic effects were evaluated on healthy cell lines and breast and colon cancer cell lines using MTT method. The results showed that the synthesized compound has high antioxidant activity. Especially the average antioxidant activity values obtained at 10 μg/mL concentration were found to be statistically significantly (*p* < 0.05) higher than the reference values of α‐TOC and BHT. When the antioxidant activity data (IC_50_) were compared separately with α‐TOC and BHT reference values, the new compound was found to be more effective. In acetylcholinesterase enzyme inhibition, the average activity values were found to be statistically significantly (*p* < 0.05) higher than the galantamine reference value. However, no statistically significant difference was observed at BChE (% inhibition) level with galantamine reference value. In terms of urease and tyrosinase enzyme inhibition activities, the urease activity of the synthesized compound was statistically significantly (*p* < 0.05) lower than the thiurea reference value. Tyrosinase activity was statistically significantly (*p* < 0.05) lower than kojic acid reference values. The synthesized and characterized compound was found to have no toxic effect on healthy cell lines and did not show any cytotoxic effect on breast cancer (MCF‐7) and colon cancer (HT‐29) cell lines.

## Graphical abstract

Antioxidant activities and IC_50_ values of KB_2_.

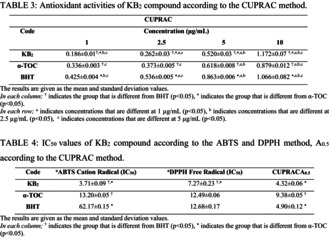

## 1 Introduction

Boron products, which have been used in the field of health for many years, have gained an important place in medical treatments due to their positive effects on cancer treatment, cardiovascular health, bone health, wound healing and the immune system [1]. Boronic acid is a type of Lewis acid. The Sp^2^ boron center has an empty p orbital that can accept a lone Lewis base pair. In the aqueous environment, boronic acids interact with a hydroxide ion, resulting in a conformational change in sp^3^ hybridized boronates. The formation of boronates depends on the concentration of the hydroxide ion. In other words, boronate formation depends on pH [2]. Boronic acids are known to have low inherent toxicity, which is one of the reasons why Suzuki coupling [3] is preferred for the development and synthesis of pharmaceuticals. Boronic acids have many uses in medicine, chemistry, biology and materials science. Since boronic acids decompose into ortho boric acid, they do not pose any environmental harm and since they are not toxic, they do not harm human health. Particularly small water‐soluble boronic acids show very low toxicity and are excreted by the kidneys without being changed. Boronic acids can be used in inflammatory diseases because they have antimicrobial and anti‐inflammatory effects [4]. 1,4‐phenyl diboronic acid used in this study has high reactivity due to the presence of the boron atom bonded to the phenyl ring [5].

Our other substance used to make ligand, 3‐methoxy catechol, is also used as an intermediate in organic synthesis and can take part in various reactions in biological systems. In some industrial applications, this catechol, used in the production of antioxidant and phenolic compounds, can function as a catalyst in some reactions in biological systems [6]. Catechol can be converted into electrophilic o‐quinones by undergoing oxidative metabolism via the cytochrome P450 enzyme system and peroxidase [7]. Also used as a precursor in pharmaceutical production, catechol is widely used in the food industry and is an important ingredient in the production of synthetic flavors such as vanillin. The first commercial vanillin synthesis began with the natural antioxidant compound eugenol, and today artificial vanillin is usually synthesized from guaiacol [8]. Additionally, some microorganisms can also produce catechol. In recent years, many studies have been conducted focusing on the biological activities of catechol and its derivatives such as 3‐methylcatechol [9].

In this research, a novel 3‐methoxy catechol boron derivative was synthesized, marking its first appearance in the scientific literature. The synthesis utilized 3‐methoxy catechol and 4‐methoxy phenyl boronic acid as precursors. This ligand was synthesized based on the hypothesis that a new compound with higher antioxidant activity could be obtained by using 3‐methoxy catechol and 4‐methoxy phenyl boronic acid, both known for their strong antioxidant activities. The resulting compound was characterized through various techniques including ^1^H and ^13^C NMR, LC–MS‐IT‐TOF, UV–Vis, and FTIR spectroscopy. Additionally, their antioxidant, anticholinesterase, antiurease, and antityrosinase activities were assessed, with several compounds demonstrating superior biological efficacy compared to standard references. Despite limited research on 3‐methoxy catechol and 4‐methoxy phenyl boronic acid in ligand synthesis, this study aims to explore their potential, particularly by identifying compounds with strong antioxidant properties that could hold promise for future health applications.

## 2 Material and Method

### 2.1 Devices Used in the Studies

FTIR‐ATR (Fourier Transform Infrared Spectroscopy, Perkin Elmer Spectrum 100), UV–Visible Spectrometer (UV–Vis absorption spectrometer, Perkin Elmer Lambda 25), ^1^H and ^13^C NMR Spectrometer (Agilent‐600 MHz ^1^H and ^13^C Nuclear Magnetic Resonance Spectroscopy), LC/MS and LC/MS‐IT‐TOF (Shimadzu LC/MS 8040 Liquid Chromatography‐Mass Spectrometry and High Speed Liquid Chromatography Ion Trap/Time of Flight Mass Spectrometry), Melting Point Determination Device (Barnstead Electrothermal 9100), Oven (Memmert), Ultra Deionized Pure Water Device (Sartorius Arium Comfort), Evaporator (Heidolph Laborato 4001 Efficient), Magnetic Stirrers (IKA C‐Mag HS‐7 and Heidolph), Elisa Reader (BioTek EON brand enzyme‐linked immunosorbent assay microplate reader), and pH meter (Mettler Toledo brand). All items were sourced from Sigma.

### 2.2 Synthesis of Compound KB_2_ (MA84) (4‐Methoxy‐2‐(4‐Methoxyphenyl) Benzo [d] [1,3,2] Dioxaborole)

A total of 0.140 g (1 mmol) of 3‐methoxy catechol was dissolved in 25 mL of THF and refluxed at 120°C. To this mixture, 0.152 g (1 mmol) of 4‐methoxy phenylboronic acid dissolved in 15 mL of THF was added, and refluxing was continued for 24 h. After completion of the reaction, the solvent was evaporated, and the resulting precipitate was recrystallized from ethanol and dried in an oven [10].

### 2.3 Methods for Determining the Biological Activity of the Obtained Compound

#### 2.3.1 Determination of Antioxidant Activity

Biological activities of the synthesized new 3‐methoxy catechol‐containing boronic acid‐derived compound: antioxidant activities were determined by ABTS cation radical scavenging activity [11], DPPH free radical scavenging activity [12] and CUPRAC methods [13].

In these assays, the reference standards employed were BHT and α‐tocopherol (α‐TOC). The anticholinesterase properties of boronic acid‐derived compound synthesized with 3‐methoxy catechol were evaluated through the inhibition of acetylcholinesterase and butyrylcholinesterase enzymes. For all antioxidant assessments, varying concentrations were utilized: 10, 25, 50, and 100 μg/mL for highly active states, and 1, 2.5, 5, and 10 μg/mL for very active states, with 100, 250, 500, and 1000 μg/mL representing inactive states. IC_50_ values were determined using graphical analysis of % inhibition against antioxidant concentrations, where IC_50_ signifies the concentration required to inhibit 50% of the initial DPPH concentration, reflecting antioxidant efficacy [14].

#### 2.3.2 Determination of Anticholinesterase Activity

Colorimetric studies were performed using the Ellman method in 96‐well microplates. This method is commonly used to assess enzymatic activity and involves measuring changes in absorbance caused by reactions with Ellman reagent sensitive to thiol groups. Each well of the microplate allows simultaneous testing of multiple samples under controlled conditions, enabling sensitive and efficient data collection for enzymatic assays [15]. The anti‐Alzheimer's activity of newly synthesized boron‐derived compound was evaluated based on their inhibition of acetylcholinesterase and butyrylcholinesterase enzymes. This was determined using a spectrophotometric method developed by Ellman and colleagues. This method is well known for its sensitivity in measuring cholinesterase inhibition, which is crucial in the search for potentially useful compounds for the treatment of Alzheimer's disease [15].

#### 2.3.3 Determination of Antiurease Activity

The antiurease enzyme activities of newly synthesized boron‐derived compound, using urease enzyme and urea as the substrate, were determined [16]. Three identical runs were performed on each sample and the results were compared for consistency and reliability.

#### 2.3.4 Determination of Antityrosinase Activity

Tyrosinase inhibition studies of synthesized boronic acid‐derived compound with 3‐methoxy catechol were performed spectrophotometrically by the Hearing Method using mushroom tyrosinase enzyme [17]. The activities of the enzyme were calculated under the same activity conditions using different concentrations of the obtained compounds (5, 10, 25, 50 μM), and the type of inhibition caused by the inhibitor at the determined inhibitor concentration according to the results obtained was determined by calculating the kinetic constants from the Lineweaver‐Burk diagram. With plate ELISA, after the first absorbance was read at 475 nm by mixing for 3 min, the same solution was kept at 37°C for 10 min, 20 μL of substrate (L‐DOPA) was added and the second reading was made at 475 nm wavelength after waiting at 37°C for 10 min again. Calculations were started with the Hearing Method.

### 2.4 Cytotoxicity Tests

#### 2.4.1 Examining Cytotoxicity With MTT Test

Healthy and cancerous cell lines of human origin were used to evaluate the cytotoxicity of boronic acid‐derived compound synthesized with 3‐methoxy catechol. Specifically, a healthy cell line derived from ATCC (PDF), a breast cancer cell line (MCF‐7) and a colon cancer cell line (HT‐29) were used. These cell lines were cultured and maintained in the laboratory under controlled conditions, including freezing, to maintain the minimum quantities required for molecular and biochemical analyses and future studies. Cytotoxicity assessments were performed using the MTT assay following the methodology outlined by Yener et al. (2019) in their study [18]. This method is widely recognized for assessing cell viability and cytotoxic effects of compounds by measuring mitochondrial activity through the conversion of MTT (3‐(4,5‐dimethylthiazol‐2‐yl)‐2,5‐diphenyltetrazolium bromide) to formazan crystals.

#### 2.4.2 Investigation of the Effects of Compound on Cell Proliferation

The effects of the synthesized boronic acid‐derived compound on the proliferation of cancer (MCF‐7 and HT‐29) and healthy (PDF) cell lines with 3‐methoxy catechol were studied using Sigma's MTT Cell Proliferation Kit. The studies were performed following protocols recommended by the company, which are standardized procedures for the assessment of cell viability and proliferation [19]. Each MTT assay was repeated 3 times and the results were checked.

### 2.5 Statistical Analysis

The data obtained was analyzed with SPSS Package Program. One sample *t*‐test was used to analyze the data. The average values obtained at each concentration level were compared with the α‐TOC and BHT reference values separately, taking into account the 0.05 alpha level. Accordingly, values less than 0.05 indicate that this difference is not a random difference, that is, the difference in question is statistically significant, while values greater than 0.05 indicate that the difference in question is not statistically significant. In case of a significant difference according to the one‐sample *t*‐test results, the findings were interpreted separately for each reference value by looking at the α‐TOC and BHT reference values. Accordingly, if the value obtained is significantly higher than the α‐TOC and BHT reference values, it is declared that the substance is active at the relevant level (e.g., 1 μg/mL), and if it is significantly lower, it is declared that the substance is not active at the relevant level.

## 3 Findings

**FIGURE 1:**
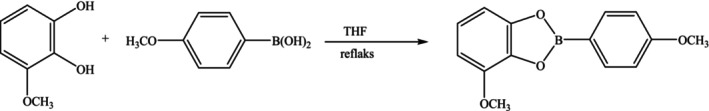
Synthesis of KB_2_.

**FIGURE 2:**
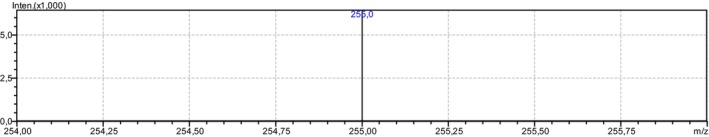
Mass spectrum of KB_2_.

**FIGURE 3:**
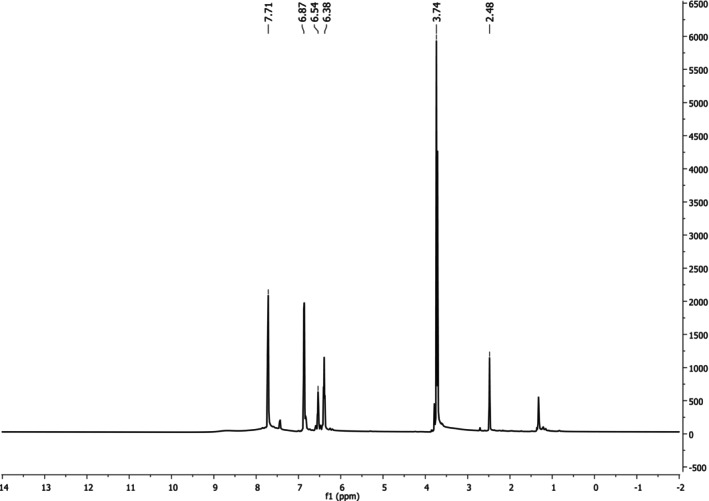
^1^H NMR spectrum of KB_2_.

^**13**^ **C NMR (ppm, EtOH‐d**_**1**_ **)**: *δ* = 103.46; 109.39; 112.88; 134.48; 136.26; 145.64; 149.12 and 161.37 (Ar‐C), *δ* = 55.97 and 55.28 (OCH_3_), *δ* = 118.10 (Ar‐C‐B). ^13^C NMR spectrum results of the KB_2_ compound are given in Figure 4.

**FIGURE 4:**
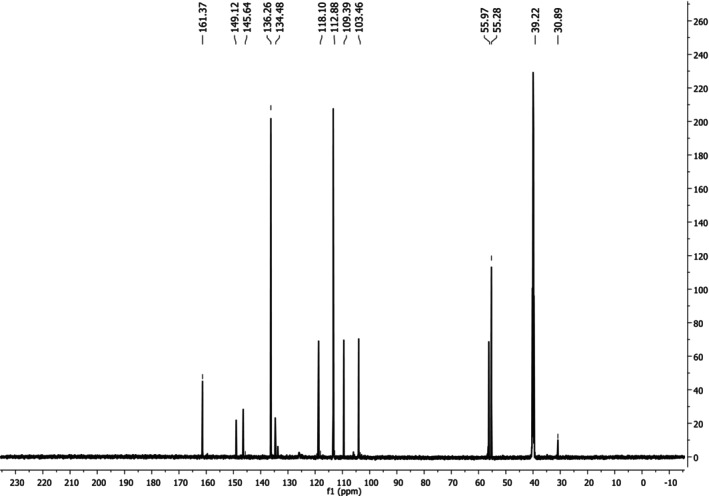
^13^C NMR spectrum of KB_2_.

**FIGURE 5:**
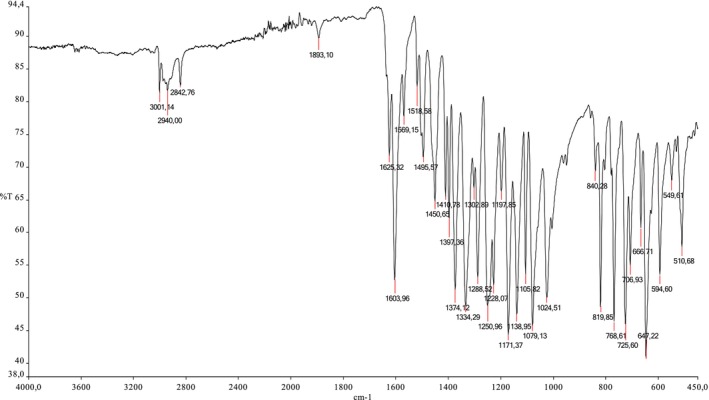
IR spectrum of KB_2_.

**FIGURE 6:**
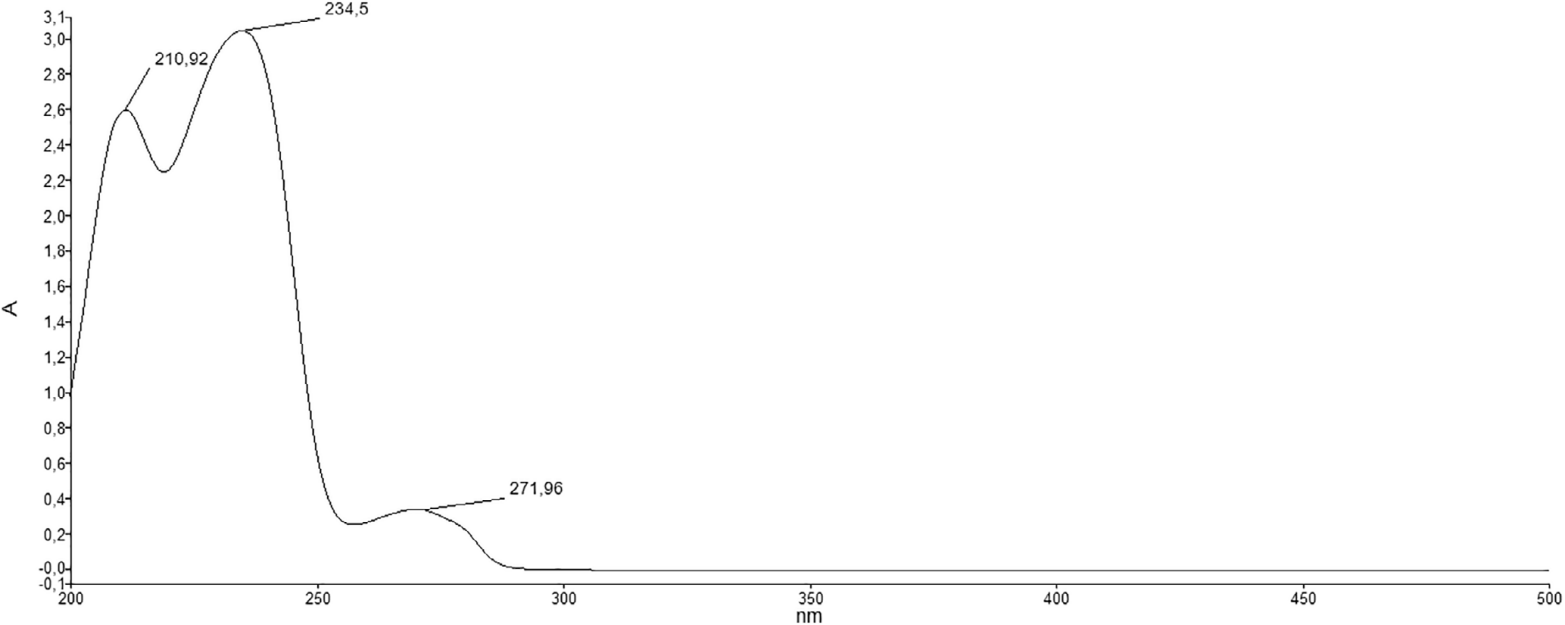
UV–Vis spectrum of KB_2_.

Findings regarding the characterization of newly synthesized compound are shown in Figures 1, 2, 3, 4, 5, 6 given in the range.

### 3.1 Findings on KB_2_ (MA 84) Compound

The synthesis scheme of the compound KB_2_ is presented in Figure 1.

Melting point: Peak observed at 87°C.

m/z: 255 [KB_2_‐H^+^]ˉ.

MW: 256.09 g/mol.

The mass spectrum of the KB_2_ compound is given in Figure 2.

^1^H NMR(ppm, EtOH‐d_1_): *δ* = 6.38–7.71 (Ar‐H), *δ* = 3.74 (OCH_3_). ^1^H NMR spectrum results of KB_2_ are given in Figure 3.

**FT‐IR (cm** ^**−1**^ **)**: 1334 and 1374 υ(BO), 1024 and 1079 υ(B‐C), 768 υ(B‐Ph), 1171 υ(Ar‐O), 1603 υ(C=C). The IR spectrum of the KB_2_ compound is given in Figure 5.

**UV–Vis (nm)**: λ_1_ = 210, λ_2_ = 234, λ_3_ = 271. UV–Vis Spectrum of KB_2_ compound is given in Figure 6.

### 3.2 Antioxidant Activity Findings of KB_2_

The findings on the antioxidant activity of the synthesized boron compound are presented in Tables 1, 2, 3, 4 below.

**TABLE 1:** Antioxidant activities of KB_2_ compound according to the ABTS method.

ABTS Cation Radical				
Code	Concentration (μg/mL)			
	1	2.5	5	10
KB_2_	15.94 ± 0.88^b^, ^c^, ^e^	46.47 ± 1.11^a^, ^c^, ^d^, ^e^	82.02 ± 3.11^a^, ^b^	86.84 ± 1.14^a^, ^b^
α‐TOC	76.47 ± 0.35	77.42 ± 0.61	78.84 ± 1.01	79.32 ± 0.89
BHT	82.59 ± 0.86	83.81 ± 0.96	84.58 ± 1.03	86.13 ± 0.97

*Note:* The results are given as the mean and standard deviation values.

^a^ Indicates concentrations that are different at 1 μg/mL (*p* < 0.05).

^b^ Indicates concentrations that are different at 2.5 μg/mL (*p* < 0.05).

^c^ Indicates concentrations that are different at 5 μg/mL (*p* < 0.05).

^d^ Indicates the group that is different from BHT (*p* < 0.05).

^e^ Indicates the group that is different from α‐TOC (*p* < 0.05).

### 3.3 Anticholinesterase Activity Findings of the KB_2_

Anticholinesterase activity values of KB_2_ compound are given in Table 5.

### 3.4 Antiurease and Antityrosinase Activity Findings of KB_2_

Antiurease and antityrosinase values of KB_2_ are given in Table 6.

### 3.5 Cytotoxicity Findings of the Synthesized Boron Compound

Cytotoxicity findings of KB_2_ are given in Table 7.

## 4 Discussion

### 4.1 Evaluation of Structure Determinations of Newly Synthesized KB_2_ Compound

The molecular weight of the newly synthesized compound KB_2_, with the molecular formula C_14_H_13_BO_4_, was determined to be 256.09 g/mol. This was confirmed through mass spectrometry, where a peak at m/z 255 corresponding to [M^+^H]^+^ was observed, indicating the compound's correct molecular formula. The intensity of this peak in the mass spectrum chromatogram, approximately 5 × 10^3^, further supported the identification and confirmation of the synthesized compound. Both molecular weight and mass spectral data conclusively aligned, verifying the successful synthesis and accurate characterization of compound KB_2_ [20, 21].

#### 4.1.1 ^1^H NMR Spectrum Results

The ^1^H NMR spectrum of the synthesized compound KB2 displayed multiple peaks between *δ* = 6.38–7.71 ppm, corresponding to aromatic hydrogen protons (Ar‐H). A single peak at *δ* = 3.74 ppm was attributed to the aromatic methoxy group (–OCH₃), providing additional confirmation of the compound's structure [22, 23].

#### 4.1.2 ^13^C NMR Spectrum Results

The ^13^C NMR spectrum revealed peaks at 103.46, 109.39, 112.88, 134.48, 136.26, 145.64, 149.12, and 161.37 ppm, which were assigned to aromatic carbon atoms. Peaks at 55.97 and 55.28 ppm were attributed to the carbon in the methoxy group, and the peak at 118.10 ppm corresponded to the carbon atom in the aromatic‐C‐B structure [24].

#### 4.1.3 IR Spectrum Results

In the IR spectrum of KB_2_, B‐O stretching was observed at 1374 cm^−1^ and 1334 cm^−1^, B‐C stretching at 1024 cm^−1^ and 1079 cm^−1^, and B‐Ph vibrations at 768 cm^−1^. Aromatic‐O stretching was indicated at 1171 cm^−1^. Peaks associated with free phenyl boronic acid were observed at 1024 cm^−1^ and 1079 cm^−1^, showing a shift from the free phenyl boronic acid peaks at 1023 cm^−1^ and 1130 cm^−1^ [24].

#### 4.1.4 Electronic Spectrum (UV) Results

The UV–Vis spectrum of KB_2_, dissolved in ethanol, revealed absorption peaks at λ_max_ = 210 nm and 234 nm, attributed to π‐π* transitions within the benzene ring. A peak at 271 nm corresponded to n‐π* transitions involving the free ‐OCH₃ group, confirming the presence of conjugated systems in the molecule and validating its structural arrangement based on absorption characteristics [25, 26].

According to all these data regarding the KB_2_ compound, it is seen that the compound has a structure as shown in Figure 1.

### 4.2 Evaluation of Biological Activity Finding of KB_2_

#### 4.2.1 Discussion of Antioxidant Activity Findings of KB_2_

When the data in Table 1 are examined, the ABTS cation radical scavenging activity of the newly synthesized and characterized KB_2_ compound; For the 1 μg/mL concentration level, the mean value obtained was compared separately with the reference values of α‐TOC and BHT. It was concluded that this average value was statistically significantly lower than the reference values of α‐TOC and BHT at the *p* < 0.05 level. Substances at a concentration of 1 μg/mL are inactive, as the average value obtained is significantly lower than both α‐TOC and BHT reference values. For the 2.5 μg/mL concentration level, the obtained value was compared separately with the α‐TOC and BHT reference values and it was concluded that the average value was statistically lower than both reference values at the level of *p* < 0.05. Since the obtained value is lower than the α‐TOC and BHT reference values, substances at a concentration of 2.5 μg/mL are inactive. For the 5 μg/mL concentration level, the obtained value was compared separately with the α‐TOC and BHT reference values and it was concluded that the average value did not differ statistically significantly from both reference values at the *p* < 0.05 level. For the 10 μg/mL concentration level, the obtained value was compared separately with the α‐TOC and BHT reference values. The average value obtained was statistically higher than the α‐TOC reference value at *p* < 0.05; However, it was concluded that this average value did not differ statistically from the BHT reference value at the *p* < 0.05 level.

For the ABTS Cation Radical (IC_50_) data in Table 4, the obtained values were compared separately with the α‐TOC and BHT reference values. The average value obtained shows that α‐TOC and BHT are statistically more active than the reference value at *p* < 0.05 level. According to the data in Table 3, the fact that all compounds of the series are active according to IC_50_ values is due to the high biological activity of boronic acid derivatives and catechol and the high activity of the resulting compound [10, 24, 27].

When the data in Table 2 are examined, the average value obtained for the 1 μg/mL concentration level in the DPPH free radical removal determination of the newly synthesized and characterized KB_2_ was compared separately with the reference values of α‐TOC and BHT. It was concluded that this average value was statistically significantly lower than the α‐TOC reference values at the *p* < 0.05 level. Since the average value α‐TOC obtained is significantly lower, substances at a concentration of 1 μg/mL are inactive. This average value does not differ statistically significantly from the BHT reference values at the *p* < 0.05 level. For the concentration level of 2.5 μg/mL, the mean value obtained was compared separately with the reference values of α‐TOC and BHT. It was concluded that this average value was statistically significantly lower than the α‐TOC reference values at the *p* < 0.05 level. Since the average value α‐TOC obtained is significantly lower, substances at a concentration of 1 μg/mL are inactive. This average value does not differ statistically significantly from the BHT reference values at the *p* < 0.05 level. For the 5 μg/mL concentration level, the obtained value was compared separately with the α‐TOC and BHT reference values and it was concluded that the average value did not differ statistically significantly from both reference values at the *p* < 0.05 level. For the 10 μg/mL concentration level, the obtained value was compared separately with the α‐TOC and BHT reference values and it was concluded that the average value did not differ significantly from both reference values at the *p* < 0.05 level.

**TABLE 2:** Antioxidant activities of KB_2_ compound according to the DPPH method.

DPPH free radical				
Code	Concentration (μg/mL)			
	1	2.5	5	10
KB_2_	7.22 ± 2.12^b^ ^–^ ^e^	15.43 ± 0.50^a^, ^c^ ^–^ ^e^	35.21 ± 1.43^a^, ^b^, ^e^	68.75 ± 0.41^a^ ^–^ ^c^
α‐TOC	32.36 ± 0.25^c^, ^d^	40.26 ± 1.32^c^, ^d^	57.85 ± 2.38^a^, ^b^, ^d^	65.41 ± 0.98^a^ ^–^ ^c^
BHT	18.85 ± 0.26^c^, ^e^	24.01 ± 0.14^c^, ^e^	36.82 ± 0.65^a^, ^b^, ^e^	63.58 ± 0.61^a^ ^–^ ^c^

*Note:* The results are given as the mean and standard deviation values.

^a^ Indicates concentrations that are different at 1 μg/mL (*p* < 0.05).

^b^ Indicates concentrations that are different at 2.5 μg/mL (*p* < 0.05).

^c^ Indicates concentrations that are different at 5 μg/mL (*p* < 0.05).

^d^ Indicates the group that is different from BHT (*p* < 0.05).

^e^ Indicates the group that is different from α‐TOC (*p* < 0.05).

For the DPPH free radical removal determination (IC_50_) data in Table 4, the obtained values were compared separately with the α‐TOC and BHT reference values. The average value obtained shows that α‐TOC and BHT are statistically more active than the reference value at *p* < 0.05 level. According to IC_50_ values, synthesized compound is active due to the high biological activity of boronic acid derivatives and catechol and the high activity of the resulting compound [10, 28].

According to the data in Table 3, in the CUPRAC method; For the 1 μg/mL concentration level, the mean value obtained was compared separately with the reference values of α‐TOC and BHT. It was concluded that this average value was statistically significantly lower than the reference values of α‐TOC and BHT at the *p* < 0.05 level. Substances at a concentration of 1 μg/mL are inactive since the average value obtained for α‐TOC and BHT is significantly lower. For the concentration level of 2.5 μg/mL, the mean value obtained was compared separately with the reference values of α‐TOC and BHT. It was concluded that this average value was statistically significantly lower than the BHT reference values at the *p* < 0.05 level. Since the average value obtained is significantly lower than BHT, substances at a concentration of 2.5 μg/mL are inactive. This average value does not differ statistically significantly from the α‐TOC reference values at the *p* < 0.05 level. For the 5 μg/mL concentration level, the mean value obtained was compared separately with the reference values of α‐TOC and BHT. It was concluded that this average value was statistically significantly lower than the BHT reference values at the *p* < 0.05 level. Substances at a concentration of 5 μg/mL are inactive since the average value obtained is significantly lower than BHT. This average value does not differ statistically significantly from the α‐TOC reference values at the *p* < 0.05 level. For the 10 μg/mL concentration level, the obtained value was compared separately with the α‐TOC and BHT reference values. The average value obtained was statistically higher than the α‐TOC reference value at *p* < 0.05; However, it was concluded that this average value did not differ statistically from the BHT reference value at the *p* < 0.05 level.

**TABLE 3:** Antioxidant activities of KB_2_ compound according to the CUPRAC method.

CUPRAC				
Code	Concentration (μg/mL)			
	1	2.5	5	10
KB_2_	0.186 ± 0.01^b^, ^c^ ^–^ ^e^	0.262 ± 0.03^a^, ^c^ ^–^ ^e^	0.520 ± 0.03^a^, ^b^ ^–^ ^e^	1.172 ± 0.07^a^ ^–^ ^e^
α‐TOC	0.336 ± 0.003^c^, ^d^	0.373 ± 0.005^c^, ^d^	0.618 ± 0.008^a^, ^b^, ^d^	0.879 ± 0.012^a^ ^–^ ^d^
BHT	0.425 ± 0.004^b^, ^c^, ^e^	0.536 ± 0.005^a^, ^c^, ^e^	0.863 ± 0.006^a^, ^b^, ^e^	1.066 ± 0.082^a^ ^–^ ^c^, ^e^

*Note:* The results are given as the mean and standard deviation values.

^a^ Indicates concentrations that are different at 1 μg/mL (*p* < 0.05).

^b^ Indicates concentrations that are different at 2.5 μg/mL (*p* < 0.05).

^c^ Indicates concentrations that are different at 5 μg/mL (*p* < 0.05).

^d^ Indicates the group that is different from BHT (*p* < 0.05).

^e^ Indicates the group that is different from α‐TOC (*p* < 0.05).

For the CUPRAC method (A_0,5_) data in Table 4, the obtained values were compared separately with the α‐TOC and BHT reference values. It can be seen that the average value obtained is statistically more active than the α‐TOC reference value at *p* < 0.05 level. For the CUPRAC(_A0,5_) value, it was concluded that the average value did not differ statistically from the BHT reference value at the *p* < 0.05 level.

**TABLE 4:** IC_50_ values of KB_2_ compound according to the ABTS and DPPH method, A_0.5_ according to the CUPRAC method.

Code	ABTS cation radical (IC_50_)	DPPH free radical (IC_50_)	CUPRACA_0.5_
KB_2_	3.71 ± 0.09^a^, ^b^	7.27 ± 0.23^a^, ^b^	4.32 ± 0.06^b^
α‐TOC	13.20 ± 0.05^a^	12.49 ± 0.06	9.38 ± 0.05^a^
BHT	62.17 ± 0.15^b^	12.68 ± 0.17	4.90 ± 0.12^b^

*Note:* The results are given as the mean and standard deviation values.

^a^ Indicates the group that is different from BHT (*p* < 0.05).

^b^ Indicates the group that is different from α‐TOC (*p* < 0.05).

Alnoman et al. (2020) synthesized a new chiral BODIPY‐based fluorescent compound [5‐bromo‐4,4‐difluoro‐3 (S)‐1‐phenylethyl)amino) BODIPY] for biomedical applications and investigated the antioxidant properties of the resulting compound. The results of the antioxidant activity of the compound, finding that its IC_50_ values are higher than ascorbic acid, support this doctoral study [29].

In conclusion, similar to the semisynthetic and fully synthetic boron derivative ligands and Schiff base boron compounds synthesized in previous studies, boronic acid derivative compounds linked with 3‐methoxy catechol show high antioxidant activity. Incorporation of 3‐methoxy catechol and boronic acid derivative in these synthesized compounds significantly contributes to their biological properties, enhancing their overall activity. These findings underscore the potential of these compounds for various biomedical applications, taking advantage of their combined antioxidant capabilities and specific biological activities observed in this study and related research [10].

#### 4.2.2 Discussion of Anticholinesterase and Butyrylcholinesterase Activity Findings of KB_2_

According to the data in Table 5, the values obtained for the acetylcholinesterase enzyme inhibition level of the newly synthesized and characterized ligand were compared separately with the galantamine reference value. It was determined that the average value obtained was statistically higher than the galantamine reference value at *p* < 0.05. For BChE (% inhibition) level, the obtained values were compared separately with the galantamine reference value. The average value obtained does not differ statistically from the galantamine reference value at the *p* < 0.05 level.

**TABLE 5:** Anticholinesterase values of KB_2_ compound.

Code	AChE (%inhibition)	BChE (%inhibition)
KB_2_	83.86 ± 3.26^a^	64.91 ± 1.24^a^
Galantamine	61.03 ± 1.46	59.51 ± 1.16

*Note:* The results are given as the mean and standard deviation values. Galantamine: satandart of AchE and BChE.

^a^ Indicates the group that is different from galantamine (*p* < 0.05).

Studies on the anticholinesterase activity of similar ligands derived from boronic acid with synthesized 3‐methoxy catechol have not been found in the literature. Semi‐synthetic boronic acid‐derived ligands synthesized in our previous studies were generally found to be active.

Sicak et al. (2020) investigated the antioxidant and anticholinesterase activities of 24 synthetic pyrazolo‐derived ligands they synthesized. They determined that compounds carrying morpholine rings were more active than compounds containing piperidinyl in both activities. They found that the anticholinesterase activity test provided higher values than galantamine in the BChE assay. For this reason, they stated that the compound 4f they synthesized could be used as an anticholinesterase substance and/or an anticholinesterase assay standard. In this study, the anticholinesterase activity results were higher than the BuChE activity results, supporting the thesis [30].

The inhibitory effects of 14 newly synthesized naphthyridine‐11‐amine derivative compounds on acetylcholinesterase (AChE) and butyrylcholinesterase (BuChE) were evaluated. The study found that 12‐(4‐Fluorophenyl)‐1,2,3,4,7,8,9,10‐octahydrodibenzo[b,g][1,8]naphthyridine‐11‐amine was the most potent AChE inhibitor, with an IC_50_ value of 0.091 μM. Additionally, 12‐(2,3‐dimethoxyphenyl)‐1,2,3,4,7,8,9,10‐octahydrodibenzo[b,g][1,8]naphthyridine‐11‐amine exhibited the strongest inhibition against BuChE, with an IC_50_ value of 0.182 μM [31].

Pouramiri et al. (2017) studied the biological potential inhibitors of acetylcholinesterases (AChE) and butyrylcholinesterase (BChE) of a series of new benzo[d] oxazole derivative (6a‐n) compounds they synthesized. In vitro studies have shown that most of the synthesized compounds are potent inhibitors of acetylcholinesterase and butyrylcholinesterase. In this study, the acetylcholinesterase and butyrylcholinesterase activities of the ligands were generally high [32].

#### 4.2.3 Discussion of Antiurease and Antityrosinase Activity Findings of KB_2_

When the urease and tyrosinase enzyme inhibition activities of the newly synthesized compound are examined according to the data in Table 6; For the urease level, the obtained values were compared separately with the thiourea reference value. It was determined that the average value obtained was statistically lower than the reference values at *p* < 0.05. For tyrosinase level, the obtained values were compared separately with the kojic acid reference value. It was determined that the average value obtained was statistically lower than the reference values at *p* < 0.05.

**TABLE 6:** Antiurease and antityrosinase values of KB_2_ compound.

Code	Urease (%inhibition)	Tyrosinase (%inhibition)
KB_2_	29.66 ± 0.03^a^	12.36 ± 0.33^b^
Thiourea	97.46 ± 2.01	
Kojic acid		75.79 ± 0.96

*Note:* The results are given as the mean and standard deviation values. Thiourea: standard substance of Urease. Kojic acid: standard substance of Tyrosinase.

^a^ Indicates the group that is different from thiourea (*p* < 0.05).

^b^ Indicates the group that is different from α‐TOC (*p* < 0.05).

It was determined that the urease and tyrosinase activities of the synthesized compound were lower than the standards. Although the urease inhibition activity of the KB_2_ compound is lower than the standard, it is seen that the tyrosinase inhibition activity (81.38 ± 0.78) is higher than the standard used kojic acid (75.79 ± 0.96).

No similar studies have been found in the literature regarding the urease and tyrosinase enzyme inhibition activities of similar synthesized ligands derived from boronic acid with synthetic 3‐methoxy catechol. Similar to the semi‐synthetic boron‐derived ligands synthesized in our previous studies, it was observed that the activities of boronic acid‐derived compounds bonded with 3‐methoxy catechol were also low [10, 24]. In recent years, studies have been conducted examining the urease and tyrosinase enzyme activities of synthetically synthesized ligands. Mojzych et al. (2017) tested the tyrosinase and urease inhibitory activities of a new series of sulfonamide derivatives of pyrazolo [4,3‐e] [1, 2, 4] triazine with their synthetically obtained chiral amino group. Evaluation of the prepared derivatives found that all of the compounds showed higher urease inhibitory activity than standard thiourea [33].

Kahriman et al. (2017), 3,5‐disubstituted‐2‐pyrazoline derivatives (4–6), their boron‐fluorine complexes (boron (3‐ (2′‐aminophenyl), 5‐ (2′−/3′−/4′) ‐pyridyl) pyrazoline, BOAPPY) (7–9) and boron 1,2′‐diazaflavone complex (BODAF) (11), respectively, starting from azachalcones (1–3) to diazaflavone (10) and found that they showed significant tyrosinase enzyme inhibition activities [34].

#### 4.2.4 Evaluation of the Cytotoxicity Findings of KB_2_

The toxic effects of the new compound derived from 3‐methoxy catechol boronic acid, whose structures were elucidated, on healthy cell lines (PDF) and their cytotoxic effects on cancerous MCF‐7 (breast carcinoma) and HT‐29 (colon carcinoma) were determined by MTT. It was found that the KB_2_ compound did not have a toxic effect on living cells and a cytotoxic effect on cancerous cells (Table 7).

**TABLE 7:** Toxic and cytotoxic values of KB_2_ compound.

Code	HT‐29 (200 ppm)	MCF‐7 (200 ppm)	PDF (200 ppm)
KB_2_	95.84 ± 1.20	95.10 ± 1.12	94.69 ± 1.07

*Note:* % viability values at 200 ppm concentration.

Similar studies on the cytotoxicity of newly synthesized synthetic 3‐methoxy catechol boronic acid‐derived ligands have not been found in the literature.

In our previous studies, we determined the toxic effects of the semi‐synthetic herbal boron compounds we synthesized on healthy cell lines (PDF) and their cytotoxic effects on cancerous MCF‐7 (breast carcinoma) and HT‐29 (colon carcinoma) using MTT, and determined that not all of the compounds synthesized showed toxic effects [10, 24].

Su et al. (2011) used a novel cell‐targeting, pH‐sensitive polymeric carrier for the delivery of the anticancer drug bortezomib (BTZ) to cancer cells and found that BTZ was easily conjugated to catechol‐containing polymeric carriers designed to be selectively taken up by cancer cells via cell surface receptor‐mediated mechanisms. They made it happen. The polymer used as the building block in this study was poly(ethylene glycol), chosen for its ability to reduce nonspecific interactions with proteins and cells. The catechol moiety has been used due to its ability to bind and release borate‐containing therapeutics, such as BTZ, in a pH‐dependent manner. Their study showed that the cancer‐targeting drug polymer conjugate significantly increased cellular uptake, proteasome inhibition, and cytotoxicity against breast carcinoma cells compared to nontargeting drug polymer conjugates. They observed that the pH‐sensitive catechol boronate binding mechanism provides a chemoselective approach to control BTZ release in targeted cancer cells, creating a concept that can be further applied to other boronic acid‐containing therapeutics to treat a wide range of diseases [35].

## 5 Conclusion and Recommendations

We synthesized and structurally characterized a novel boronic acid derivative incorporating 3‐methoxy catechol and conducted comprehensive biological activity evaluations. The compound demonstrated notable antioxidant activity and exhibited inhibition of acetylcholinesterase, urease, and tyrosinase enzymes, along with cytotoxic effects in both normal and cancerous cell lines. It was particularly effective in inhibiting acetylcholinesterase compared to butyrylcholinesterase.

Given the novelty of the synthesis, direct comparisons with similar boronic acid compounds were limited. However, the promising biological activities observed in KB2 align with findings in the literature on boron‐containing complexes, particularly their selective cytotoxicity against cancer cells while sparing healthy ones. The integration of boronic acid derivatives and 3‐methoxy catechol was hypothesized to enhance biological activities, which was supported by our experimental results. This combination led to significant antioxidant and enzyme inhibitory effects, along with selective cytotoxicity, suggesting its potential as a therapeutic agent.

The observed bioactivity of this 3‐methoxy catechol boronic acid derivative highlights its potential for medicinal chemistry, especially in the development of novel cancer treatments or active pharmaceutical ingredients. Moreover, the compound shows promise for creating bioactive substances that could be utilized in the food and cosmetic industries.

In conclusion, the synthesized compound demonstrates significant therapeutic and industrial potential, warranting further exploration and development in various bioactive applications.

## Author Contributions

Hamdi Temel was responsible for conducting all experimental studies, as well as writing and interpreting the article. Emine Baydan contributed to the writing, interpretation, and guidance of the article.

## Conflicts of Interest

The authors declare no conflicts of interest.

## Data Availability

Data will be made available upon request from the corresponding author.
